# The efficacy of electroacupuncture for postherpetic neuralgia arising from herpes zoster affecting the cephalo-facial area: study protocol for a double center randomized controlled trial

**DOI:** 10.3389/fmed.2025.1616035

**Published:** 2025-08-28

**Authors:** Pengfei Qiu, Haiju Sun, Yunfan Xia, Siying Qu, Jianqiao Fang, Xiaoyu Li

**Affiliations:** ^1^Department of Acupuncture and Moxibustion, Zhejiang Hospital, Hangzhou, Zhejiang, China; ^2^Department of Acupuncture and Moxibustion, The First Affiliated Hospital of Zhejiang Chinese Medical University (Zhejiang Provincial Hospital of Traditional Chinese Medicine), Hangzhou, Zhejiang, China; ^3^Department of Acupuncture and Moxibustion, The Third Affiliated Hospital of Zhejiang Chinese Medical University (Zhongshan Hospital of Zhejiang Province), Hangzhou, Zhejiang, China; ^4^Department of Neurobiology and Acupuncture Research, The Third School of Clinical Medicine, Key Laboratory of Acupuncture and Neurology of Zhejiang Province, Zhejiang Chinese Medical University, Hangzhou, China

**Keywords:** postherpetic neuralgia, electroacupuncture, pain, protocol, randomized controlled trial

## Abstract

**Introduction:**

Postherpetic Neuralgia (PHN) constitutes a severe sequelae following herpes zoster (HZ) Infection, and one of the most problematic issues is the treatment of cephalo-facial PHN in patients over 50 years of age, which severely affects the patient’s work mood, sleep and activities of daily living. The efficacy of conventional treatments for PHN remains unsatisfactory. Therefore, there is an urgent need for alternative approaches to explore simpler, more convenient, effective, and inexpensive treatment options in the clinical treatment of PHN. This trial aims to thoroughly evaluate the effectiveness and safety of EA as a therapeutic modality for individuals suffering from cephalo-facial PHN.

**Methods and analysis:**

The protocol outlines a double-center, randomized, and controlled trial design where both patients and assessors are blinded to the intervention being administered. The duration of the trial’s therapeutic intervention will span 4 weeks, followed by a 2-month observation period for monitoring any subsequent effects or outcomes. The 124 qualified individuals will be randomly allocated in a balanced 1:1 ratio to either the EA group or the drug group. All variables will undergo evaluation at the start of the study (week 0, baseline), during the treatment period at weeks 2 and 4, and during the follow-up period at weeks 8 and 12. The primary outcome is the Visual Analog Scale (VAS). Secondary outcomes include the Brief pain inventory-Facial scale (BPI-Facial), Pittsburgh Sleep Quality Index Scale (PSQI), Self-rating depression scale (SDS), Hamilton depression scale (HAMD), and Quality of Life Rating Scale (SF-36). The occurrence of any adverse reactions will be monitored and assessed throughout the duration of the trial.

**Conclusion:**

This study will preliminarily evaluate the efficacy and safety of electroacupuncture (EA) in the treatment of patients with postherpetic neuralgia (PHN).

**Ethics and dissemination:**

Ethical approval for this trial has been obtained from the Institutional Ethics Review Board of the Third Affiliated Hospital of Zhejiang Chinese Medical University (No. ZSLL-KY-2023-029-01) and Zhejiang Hospital (No. 2024-030-K). Before enrollment, participants will be required to sign a form of informed consent.

**Clinical trial registration:**

Identifier NCT06420778, https://clinicaltrials.gov/study/NCT06420778.

## Introduction

Postherpetic neuralgia (PHN) refers to a type of pain that persists for a duration of 1 month or longer, following the healing of an acute herpes zoster (HZ) rash (1). Epidemiological studies have shown that the occurrence rate of HZ in Europe, North America and the Asia Pacific region stands at 5 to 6 cases per 1,000 individuals per year and up to 30% of HZ patients develop PHN (2, 3). In China, the rate of PHN incidence is rising steadily each year, and the incidence increases with age (4). Clinical studies have found that PHN occurs mostly in the elderly, where the occurrence rate of PHN among patients aged over 50 can reach as high as 60 to 75%, which has a significant impact on both physical and mental well-being, as well as the overall quality of life (5, 6).

PHN, as a pain disorder that severely affects the quality of human life, faces many clinical challenges. One of the most problematic issues is the treatment of cephalo-facial PHN in patients aged 50 and above. The clinical challenges of cephalo-facial PHN in patients over 50 years of age are more daunting (7, 8), making the clinical symptoms of cephalo-facial PHN in patients over 50 years of age more complex and more difficult to treat. Therefore, this project will focus on patients over the age of 50 with cephalo-facial PHN for research.

Currently, the main clinical treatment options for PHN include drug therapy (9–11) and surgical treatment (12, 13) and alternative medicine (14, 15). Pregabalin has proven efficacy in the treatment of PHN (16) and is classified as a first-line clinical medication by guidelines (17); therefore, this study will use pregabalin as a control to investigate the clinical efficacy and safety of EA in patients with PHN. However, all of these treatment options have limitations. Studies have shown that 81.82% of patients with PHN still suffer from a similar degree of pain as before treatment, and their probability of adverse drug reactions is as high as 42.74% (18, 19). Although surgical treatment is effective for PHN, there is a greater risk of irreversible damage to the nerves and complications for patients over 50 years of age with cephalo-facial PHN (20). Therefore, there is an urgent need for alternative approaches to explore simpler, more convenient, effective, and inexpensive treatment options in the clinical treatment of cephalo-facial PHN over 50 years of age.

In the last decade, domestic and international clinical studies and evidence-based medicine suggest that complimentary medicine serves as an effective treatment option for PHN with minimal side effects (21–23). In the current acupuncture treatment program, most of the acupuncture treatments, such as milli-acupuncture, fire-acupuncture, and electro-acupuncture combined with other therapies are chosen (24, 25). Although the effectiveness of acupuncture in the therapy of PHN has been widely recognized, there is a variety of clinical therapies and a lack of uniform standardized treatment protocols (14, 26, 27). Previous clinical studies have focused more on PHN occurring in the body, and few acupuncture treatment protocols have been reported for PHN in the head and face.

Our scientific team has long been dedicated to the in-depth clinical practice and fundamental scientific research of acupuncture therapy in the field of relieving and managing neuropathic pain (28, 29). Previous studies conducted by us have shown that acupuncture, particularly EA, is efficacious in relieving neuropathic pain (30–34). It has been shown that the mechanism of EA analgesia is related to the interaction of a number of biologically active chemicals (e.g., opioids, serotonin, norepinephrine, etc.) in the periphery, spinal cord, and higher central nervous system (35). Therefore, EA may be one of the effective means of treating PHN of the head and face.

We has used EA to treat classical trigeminal neuralgia with satisfactory efficacy (36). Moreover, the treatment of PHN with EA has achieved preliminary efficacy in the clinic (37, 38), but no systematic study has been conducted, which is not conducive to the formation of a high-quality clinical expert consensus and hinders its clinical application and further promotion.

Therefore, this project intends to evaluate the efficacy of EA therapy for PHN patients from the perspective of clinical needs, using a randomized controlled trial with oral pregabalin as the control group, and adopting the evaluation index system acknowledged both domestically and internationally, as well as evaluating the safety and the impact on the patients’ quality of life, so as to further clarify the advantages of the program in the treatment of cephalo-facial PHN, in terms of immediate efficacy and aftereffect, and thus laying a foundation for further promotion and providing an innovative, evidence-based, and efficient approach for the clinical analgesia of PHN.

## Method and analysis

### Study design

**Figure 1:**
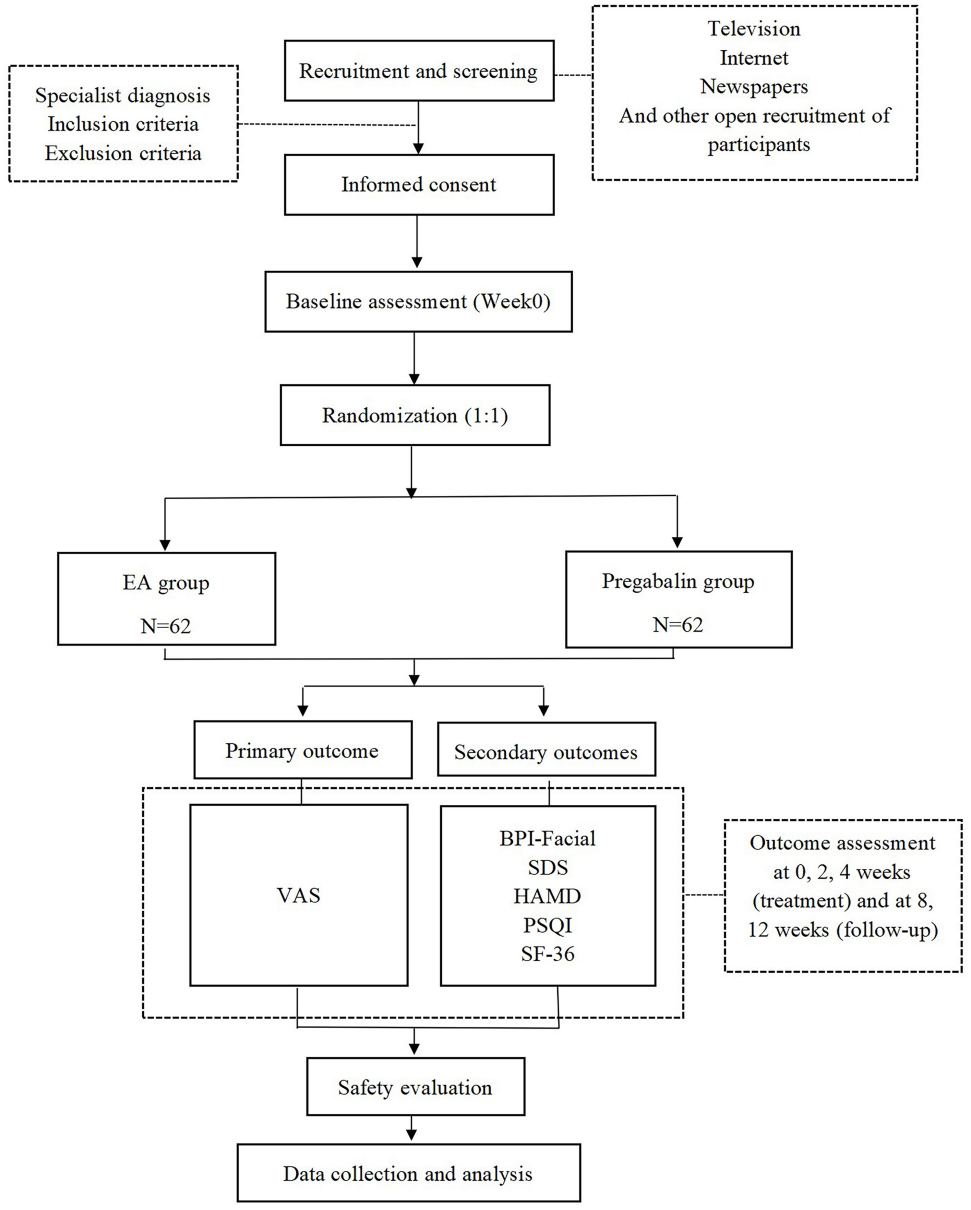
Flow chart of the study process. EA, electroacupuncture; VAS, Visual Analogue Scale; BPI-Facial, Brief Pain Inventory-Facial Scale; SDS, Self-Rating Depression Scale; HAMD, Hamilton Depression Scale; PSQI, Pittsburgh Sleep Quality Index Scale; SF-36, Short Form 36 Health Survey.

This research is structured as a randomized, parallel-group, controlled clinical trial with patient-evaluator blinding. Based on the inclusion and exclusion criteria, patients with PHN of the head and face who met the requirements will be selected as the test subjects and randomly assigned to the EA group or the drug group. The efficacy of the subjects in the EA group and the drug group will be observed before treatment, immediately after the first treatment, after 2 weeks of treatment, after 4 weeks of treatment, at 1-month follow-up after the end of treatment and at 2-month follow-up after the end of treatment, respectively. The trial design is summarized in Figure 1, and the trial schedule for enrollment, treatment, and assessment is shown in Table 1. The reporting framework for this protocol adheres to the rigorous Standards for Reporting Interventions in Clinical Trials of Acupuncture (STRICTA) (39) as well as the comprehensive guidelines outlined in the Standard Protocol Items: Recommendations for Interventional Trials (SPIRIT) (40), ensuring a thorough and transparent documentation of the study.

**Table 1:** Schedule of enrolment, treatments, and assessments.

Study period	Enrolment/baseline	Treatment period		Follow-up period	
Assessment point	1	2	3	4	5
Time	−2 Weeks to 0	2 Weeks ± 3 Days	4 Weeks ± 3 Days	8 Weeks ± 3 Days	12 Weeks ± 3 Days
Eligibility screening	√				
Demographic data	√				
Case data	√				
Inclusion criteria	√				
Exclusion criteria	√				
Informed consent	√				
Treatment		√	√		
Outcome assessment					
(1) VAS	√	√	√	√	√
(2) BPI-Facial	√	√	√	√	√
(3) SDS	√	√	√	√	√
(4) HAMD	√	√	√	√	√
(5) PSQI	√	√	√	√	√
(6) SF-36	√	√	√	√	√
Safety assessment (EA)		√	√		
Safety assessment (Medication)		√	√		
Adverse events	√				

EA, electroacupuncture; VAS visual analog scale, BPI-Facial Brief pain inventory-Facial scale; SDS Self-rating depression scale; HAMD Hamilton depression scale; PSQI Pittsburgh Sleep Quality Index Scale; SF-36, Short Form 36 Health Survey.

### Participant enrollment

Participants are being recruited mainly from the Third Affiliated Hospital of Zhejiang Chinese Medical University and Zhejiang Hospital. Subject recruitment is carried out through multiple channels, with researchers focusing on following up with specialty outpatient clinics and regular screening of hospitalized patients, and active communication with patients. In addition, measures such as paper media and WeChat are regularly taken to recruit for the whole society.

### Eligibility criteria

Expert physicians will base their diagnosis of PHN on a comprehensive assessment that includes the patient’s medical history, reported symptoms, the visible characteristics of distinctive lesions, and their distribution along a single dermatome. The specific criteria for eligibility are outlined below.

#### Inclusion criteria

1. Meets the diagnostic criteria in the 2016 Chinese Expert Agreement on Diagnosis & Treatment of PHN (41);
2. History of herpes zoster of the head and face with healed and resolved head and face lesions, neuralgia symptoms persisting ≥1 month after lesion healing, baseline facial pain profile score ≥4, and abnormal sensation and pain sensitivity of the skin around the lesions;
3. 50 ≤ Age≤80 years, gender not limited;
4. Consciousness, pain perception and discrimination, able to complete fundamental communication; and
5. Participants who have willingly given their informed consent to join this research.

#### Exclusion criteria

1. Herpes zoster occurs on the perineum, or in special types such as visceral shingles, meningeal shingles, and generalized shingles;
2. Those who take a daily oral pregabalin dose of less than 0.2 g or more than 0.6 g for analgesia prior to inclusion;
3. Patients who have had a serious adverse reaction to taking Prevacid, are allergic to acupuncture, or fall within the range of contraindications to electroacupuncture;
4. Those who have a combination of severe heart, liver, or kidney damage, epilepsy, head injury, or other major illnesses such as cognitive dysfunction, aphasia, or psychiatric disorders, and who are unable to cooperate with treatment;
5. Comorbidity with poorly controlled hypertension and diabetes mellitus;
6. Patients who are pregnant or breastfeeding; and
7. Patients who are participating in other studies and have been enrolled in other clinical studies patients within the past 3 months.

#### Discontinuation criteria

1. A physician responsible for diagnosis assesses exacerbations of PHN occurring during the study, and when a patient experiences facial pain symptoms that persisted despite acupuncture treatment, as indicated by a VAS score exceeding 8, the physician evaluates the severity and subsequently discontinues the study;
2. The specialist assesses the occurrence of significant adverse reactions during the study to make a decision on whether to proceed or discontinue the research;
3. Participants who encounter severe complications or illnesses during the study that necessitate urgent medical attention; and
4. Participants who have additional reasons that prevent them from continuing with this study.

#### Elimination criteria

1. Self-withdrawal or loss of visits by subjects;
2. Those who have not reached 2 treatments; and
3. Those who experience serious adverse reactions or adverse events during treatment.

(Note: Subjects who are dislodged should be accounted for, and their study medical records should be retained for review and not included in the final statistical analyses.)

### Randomization and allocation concealment

The envelope randomization method will be used for randomization. Upon meeting both the inclusion and exclusion criteria, subjects will be enrolled into the study. The randomization personnel or clinical researchers will randomize them based on the pre prepared patient grouping envelopes. The randomization protocol for this study will be generated by the Clinical Evaluation Center at the subject’s institution, and this person will be not involved in the statistical analysis of this project. The randomization scheme for this study and the individual parameters set in the process of generating that scheme are collectively referred to as the blind bottom, which is sealed and signed by the person generating the randomization scheme and kept by a dedicated subject matter manager not involved in this project. Participants in subgroups are not involved in the statistical analysis of data. The evaluation of the efficacy indicators will be collected and organized by a person who is unaware of the subgroups, with a triple separation of the researcher, the acupuncture operator, and the statistician.

### Blinding

Because this project is a needling treatment, the needling operator must be in direct contact with the patient, to whom blindness cannot be applied. However, blinding is implemented for all other personnel involved in the study (including patients, those who recorded and entered the observational indicators, and the data statisticians), who will be unaware of the specific grouping of patients. To guarantee a more effective execution of the blind approach, each patient will be treated individually as much as possible, minimizing the need for patients to discuss with each other about the acupuncture treatment method, their feelings during the acupuncture, and the effects of the treatment.

### Intervention

Subjects will undergo either EA or pharmacotherapy. Both groups will receive treatment for a duration of 4 weeks, followed by a 8-week follow-up period.

### Shallow plexus acupuncture combined with EA

The patients’ needles are uniformly used Huatuo brand disposable acupuncture needles sourced from Suzhou Medical Supplies Factory Co., Ltd., with specifications of 0.18 × 25 mm (needles for local acupoints) and 0.25 × 40 mm (needles for distal acupoints); and electro-acupuncture instruments are uniformly used with Han’s Acupoint Nerve Stimulation Instrument (model HANS-200). Acupuncture treatment is operated by an acupuncturist above the level of attending, and care is taken to sterilize the operator’s hands before and after the treatment, as well as to disinfect the local skin.

#### Prescription for acupuncture treatment

The main local acupoints will be chosen to be Sibai (ST2), Xiaguan (ST7), Dicang (ST4), Cuanzhu (BL2) and a-shi point on the affected side. Select acupoints according to the branch of the lesion, for eye branch lesions, Tongziliao (GB1) will be selected; for maxillary branch lesions, Quanliao (SL18) will be selected; for mandibular branch lesions, Jiache (ST6) will be selected. The distal acupoints will be chosen bilaterally at Hegu (L14), Waiguan (TE5), Taichong (LR3), and Sanyinjiao (SP6). For the specific positioning of acupuncture points, refer to the 2006 National Standard (GB/T12346-2006) of the People’s Republic of China, “Names and Positioning of Acupoints.” Table 2 provides a concise overview of the acupoint locations employed in this trial.

**Table 2:** Indication and localization of acupoints for the management of PHN.

Acupoints	Location
Primary acupoints	
A Shi acupoints	A Shi points refer to the specific locations within the herpes zoster lesion that experience the most intense pain sensations.
Si Bai (ST2)	Si Bai is on the face, with the eye looking straight down and the pupil straight down, when the infraorbital foramen is in the depression.
Xia Guan (ST7)	Xia Guan is on the face, in the depression between the center of the lower edge of the zygomatic bone and the mandibular incision.
Di Cang (ST4)	Di Cang is on the face, and is located in the depression formed by the zygomatic arch and the mandibular incision.
Cuan Zhu (BL2)	Cuan Zhu is on the face, in the depression of the eyebrow, at the supraorbital notch.
Supplementary acupoints	
Tong Zi Liao (GB1)	Tong Zi Liao is on the face, next to the lateral canthus of the eye, when the outer edge of the orbit.
Quan Liao (SL18)	Quan Liao is on the face, when the outer canthus of the eye is straight down, in the depression of the lower edge of the zygomatic bone.
Jia Che (ST6)	Jia Che is on the cheek, about 1 horizontal finger in front of and above the angle of the lower jaw, when the biting muscle bulges when chewing, and the depression is pressed.
He Gu (L14)	He Gu is situated in the area between the first and second metacarpal bones, precisely at the midpoint along the radial (thumb-side) border of the second metacarpal bone.
Wai Guan (TE5)	Wai Guan is positioned on the line extending from Yangchi point to the tip of the elbow, located 2 cun above the transverse crease of the wrist, situated between the ulna and radius bones.
Tai Chong (LR3)	Tai Chong is on the dorsal side of the foot, in the depression just before the union of the first and second metatarsal bones.
San Yin Jiao (SP6)	San Yin Jiao is positioned on the inner side of the lower leg, 3 cun above the prominence on the inner ankle bone, located along the posterior edge of the medial aspect of the tibia bone.

#### Operation

The patient is laid down in a supine posture, the whole body is relaxed, and the facial skin is routinely sterilized. Local points along the head and face herpes zoster lesion branch with 0.18 × 25 mm millimeter needle series of shallow stabbing method row stabbing method. Localized points do not necessarily require a sense of “getting qi,” the needle should be performed lightly, do not touch the trigger point. The distal acupoints of L14 and TE5 can be stabbed directly with 0.25 × 40 mm millimeter needles (to a depth of 20–30 mm), and after the patient consciously feels localized soreness and distension (“getting qi”), twisting and lifting insertion and diarrhea can be performed 10 times. If the patient is in a period of persistent pain, the first acupuncture points in the distal tract (L14 and TE5 points) can be needled, applying a large twisting, lifting and inserting diarrhea, and then needling the local acupoints after the pain has been slightly relieved.

#### EA parameters

Han’s Acupoint Neurostimulator (model HANS-200) is selected, local acupoints are chosen as ST7 + SL18 or ST7 + ST6, and distal acupoints were chosen as L14 + TE5 connected to EA, with sparse and dense waveforms of 2/100 Hz, and the duration of the treatment is 60 min, and the current intensity is tolerated by the patient.

#### Course of treatment

The needles are retained for 60 min during each time, which occurs every other day, resulting in a treatment schedule of 3 times per week for 4 consecutive weeks, amounting to a total of 12 treatments.

#### Medication group

In this group, participants undergo treatment through the oral ingestion of pregabalin capsules (specification: 75 mg*8 capsules, manufactured by Pfizer Pharmaceutical Co., Ltd.) only, 150 mg each time, twice a day, for 4 consecutive weeks.

### Outcome measures

#### Primary outcome measure

The Visual Analog Scale (VAS) allows patients to self-assess the intensity of head and facial pain by marking a point on a graduated straight line, ranging from 0 (indicating no pain) to 10 (representing the worst intolerable pain), with higher scores indicating greater pain severity. To elaborate, a score of 0 signifies the absence of pain; scores ranging from 0 to 3 denote mild and manageable pain; scores between 4 and 6 indicate pain that interferes with sleep yet remains tolerable; while scores from 7 to 10 represent severe and intolerable pain that significantly disrupts sleep or other daily activities. It is the most widely utilized tool for evaluating subjective symptoms, particularly in measuring pain severity (42, 43).

#### Secondary outcome measures

Secondary outcome measures include the Brief pain inventory-Facial scale (BPI-Facial), Pittsburgh Sleep Quality Index Scale (PSQI), Self-rating depression scale (SDS), Hamilton depression scale (HAMD), and Quality of Life Rating Scale (SF-36).

The BPI-Facial is a specialized assessment tool tailored for facial pain, covering aspects such as pain intensity, frequency of episodes, duration, and the extent to which pain interferes with daily functions like eating, speaking, and emotional well-being. Its primary role is to quantitatively evaluate the severity of facial pain and its impact on patients’ quality of life, providing valuable reference for the diagnosis and treatment of facial pain-related conditions (36, 44). During administration, respondents rate each item based on their personal experiences (e.g., pain intensity is assessed using a 0–10 numerical rating scale). The composite scores enable a comprehensive assessment of facial pain in patients with postherpetic neuralgia of the head and face, with higher scores indicating more severe pain and greater disruption to quality of life.

The PSQI is a standardized self-rating questionnaire designed to assess sleep quality over the past month. It encompasses seven key dimensions, including sleep latency, sleep duration, sleep efficiency, and others. The scale quantifies subjective sleep experiences through 19 questions and is widely utilized in clinical and research settings for screening and evaluating sleep disorders (45). During administration, respondents independently complete the self-assessment items, with scores calculated for each of the seven components (ranging from 0 to 3 points per component). The total score ranges from 0 to 21, with higher scores indicating poorer sleep quality.

The SDS is a self-assessment tool designed to evaluate depressive states in adults, comprising 20 items that address depression-related symptoms such as low mood, diminished interest, sleep disturbances, and appetite changes. Its primary function is to rapidly screen for the presence and severity of depressive symptoms, providing valuable reference for clinical diagnosis and psychological evaluation (25). During administration, respondents rate each item based on their actual experiences over the past week using a 4-point Likert scale (1–4 points). The raw scores of all items are summed to obtain a total score, which is then converted into a standardized score to determine the level of depression (below 53: normal; 53–62: mild depression; 63–72: moderate depression; above 72: severe depression).

The HAMD is a widely used clinician-administered tool for assessing the severity of depressive symptoms (46). It is evaluated by trained professionals through patient interviews and clinical observations. The scale encompasses over 20 symptom domains, including depressed mood, feelings of guilt, suicidal ideation, sleep disturbances, loss of interest in work, psychomotor retardation, agitation, anxiety, and others, with each item rated on a 5-point Likert scale (0–4). Higher total scores indicate greater severity of depressive symptoms, with typical classifications as follows: below 7 points suggesting no depression, 7–17 points indicating mild depression, 18–24 points representing moderate depression, and scores above 24 denoting severe depression.

The SF-36 is a widely utilized generic self-administered questionnaire for assessing health-related quality of life (HRQoL). It comprises 36 items covering eight dimensions: physical functioning, role-physical, bodily pain, general health perceptions, vitality, social functioning, role-emotional, and mental health. Its primary purpose is to comprehensively evaluate an individual’s physiological, psychological, and social functional status, and it has been extensively applied in evaluating HRQoL and intervention outcomes among patients with postherpetic neuralgia (47, 48). During administration, respondents answer based on their actual conditions over the past 4 weeks. Scores for each dimension are calculated using standardized scoring algorithms and transformed into a 0–100 scale, with higher scores indicating better health status in that specific dimension.

Patients with PHN are usually plagued by chronic pain that can somewhat affect sleep and mood, thus sleep quality and mood state can in turn affect their pain intensity, therefore the PSQI, SDS, HAMD and SF-36 will be used to assess the participants’ mental status and sleep quality. Outcome Measures will be performed at the baseline (pre-treatment), the 2 week after intervention, the 4 week after intervention, the 1-month follow-up and the 2-month follow-up to evaluate the severity of the disease and the treatment effect.

### Safety evaluation

Relevant safety indicators are recorded on the Subject Adverse Event Record Form by the acupuncturist at any time during treatment and follow-up:

#### Safety evaluation related to EA

Broken needles, missed needles, needle fainting, unbearable acupuncture-induced pain (VAS ≥ 8 points), localized hematomas, infections, and abscesses; symptoms, mean degree, and mean duration of other post-acupuncture discomfort (encompassing pain, dizziness spells, anorexia, headache, and insomnia that occurred ≥1 h in duration after acupuncture).

#### Pregabalin adverse reactions

Distorted or overlapping vision, sleepiness, stomach discomfort, exhaustion, neuropsychiatric disturbances, other.

### Quality control and data management

First of all, the research group will formulate the unified Standard Operating Procedures (SOPs), and prior to the official commencement of the clinical trial by 1 month, the research team will organize a dedicated training session aimed at imparting standardized training to all researchers participating in the study. The core emphasis of the training revolves around the project’s execution plan and the adherence to various SOPs. This is to ensure that every clinical researcher is thoroughly acquainted with the research methodology, including the intricate implementation details, thereby guaranteeing the credibility and authenticity of the clinical research findings.

Furthermore, all observations recorded during clinical studies must undergo rigorous verification and multiple rounds of confirmation to uphold the reliability and authenticity of the data. This approach ensures that every result and conclusion presented in clinical studies is derived solely from the primary, unadulterated data, maintaining the integrity of the research outcomes. Expert data administration companies are commissioned to conduct clinical data management, and monthly clinical research quality checks are strictly implemented. Reasons for withdrawal will be systematically recorded for all participants who discontinue the trial at any stage, and the resulting dropout rate will be statistically analyzed.

### Sample size estimation

This research involves a randomized controlled trial design, where eligible participants will be allocated to two distinct groups in an equal proportion of one participant to another (1:1 ratio), and the primary outcome metric is the percentage of cases with a pain intensity VAS ≤ 2 points after 4 weeks of treatment. According to the project team’s pre-efficacy observation pre-trial, the effective rate of this study’s treatment regimen in treating PHN is about 91%, and the literature reported that the effective rate of pregabalin is 74.4% (49). The estimation of the required sample size formula for the test of superiority of the independent sample rate of two groups is used for the calculation, taking *α* = 0.025 and *β* = 0.2, with the same number of sample cases between the groups, and using a one-sided test, taking into account a 20% dropout rate. Each group will necessitate a minimum of 62 cases, resulting in a combined total of 124 cases across the two groups.


$$N=\frac{2{\left[Z_{\alpha /2}\sqrt{2\bar{p}\bar{q}}+Z_{\beta }\sqrt{p_{0}q_{0}+p_{1}q_{1}}\right]}^{2}}{{\left(p0-p1\right)}^{2}}$$

### Statistical analysis

The statistical analysis of the data will be independently conducted by a third-party statistician, unaffiliated with the pre-test phase, utilizing the SPSS 25.0 software. Measurements are described as mean ± standard deviation ( $\bar{x}\pm s$ ), median, maximum, minimum, and quartiles, and counts are expressed as percentages (%). All hypothesis tests will be conducted using a two-tailed approach, with statistical significance being determined at a threshold of *p* < 0.05. Baseline data will be evaluated for between-group comparability with a two-tailed statistical test at the *α* = 5% level. For count data, the chi-squared test will be employed to analyze differences between groups. For measured data, the t-test will be utilized to compare groups. And for nonparametric variables, the Wilcoxon rank-sum test will be adopted to assess group comparisons. Baseline data will be compared between groups with two-tailed statistical tests at the α = 5% level. If the data conform to normal distribution and is chi-square, t-test for independent samples will be employed to compare differences between groups, whereas a paired t-test will be utilized to assess changes within the same group over time or under different conditions. The chi-square test will be used for count data, and nonparametric tests will be used for comparison of rank data and data not conforming to normal distribution. At a significance level of *α* = 0.05, a *p*-value less than 0.05 (*p* < 0.05) suggests that the observed difference is statistically significant. After all data entry and checking are completed, the statisticians will carry out the statistical analysis work in a timely manner and issue a written statistical analysis report.

### Ethical approval and study registration

Ethical approval for this trial has been obtained from the Ethics Panel of the Third Affiliated Hospital of Zhejiang Chinese Medical University (Approval File: ZSLL-KY-2023-029-01) and Zhejiang Hospital (No.2024-030-K). Prior to enrollment, participants will be comprehensively apprised of the trial’s objectives, study benefits, and potential risks. Prior to engaging in this trial, it is mandatory for participants to provide their written informed consent, empowering them with the full autonomy to choose whether they wish to participate or not. To protect the participant’s privacy, the case report form will document the participant’s age, and additionally, the participant’s initials will be included on the form for identification purposes. The research protocol has been officially recorded in the Clinical Trials Registry, assigned with a unique identification number (NCT6420778) for tracking purposes.

## Discussion

PHN, as a pain disorder that seriously affects the quality of human life, faces many clinical challenges. One of the most problematic issues is the treatment of cephalo-facial PHN in patients aged 50 and above (7, 50). Currently, treatment mainly focuses on medication and surgery, but the side effects are so great that patients are unable to adhere to them for a long time or be cured completely. Therefore, there is an urgent need to explore simpler, more convenient, effective, and inexpensive treatment options. The treatment of cephalo-facial PHN with electroacupuncture has a certain clinical efficacy, but lacks the support of systematic clinical research results, which is not conducive to the popularization and promotion of a wide range. Therefore, there is an urgent need for a randomized controlled trial with rigorous methodological quality to further investigate the efficacy and safety of electroacupuncture in the treatment of PHN.

In recent times, numerous prestigious publications have documented the positive impact of acupuncture in managing pain-related conditions (51–54). Findings from a substantial clinical study indicate that acupuncture could markedly alleviate pain and possibly enhance survival rates among breast cancer patients (53). EA is a commonly used therapeutic method for clinical analgesia in acupuncture, and domestic and international researchers have preliminarily affirmed the analgesic effect of EA on PHN (37, 38, 55). According to reports (56–58), studies have shown that EA exerts an inhibitory influence on pain through the interaction and integration of acupuncture signals with pain signals at various levels of the nervous system. This process involves numerous bioactive substances, including serotonin, gamma-aminobutyric acid (GABA, opioid peptides, TRPV1 and P2X3), and so on. For the tonic and laxative effects of EA, it is currently believed that low-frequency EA has an excitatory effect to supplement deficiency; high frequency EA has antispasmodic and analgesic effects, used for purging excess; and the 2/100 Hz dense wave EA has both the effect of tonifying deficiency and reducing excess, which is used to flatten deficiency and reduce diarrhea. Patients with cephalo-facial PHN have severe pain, and their treatment principle should be “tonifying deficiency and removing blood stasis.”

In addition, the VAS is selected as the primary outcome measure for evaluating EA efficacy in treating cephalo-facial PHN, primarily due to its ability to continuously and quantitatively capture dynamic changes in pain intensity. VAS provides high sensitivity in reflecting patients’ subjective pain experiences, with operational simplicity that facilitates the detection of both immediate and cumulative analgesic effects induced by EA. As an internationally validated pain assessment tool, VAS aligns closely with the neurophysiological mechanism of EA (modulating neural signal transmission), and its score changes directly validate the achievement of the primary therapeutic goal (pain relief). This offers a standardized basis for clinical decision-making and inter-study efficacy comparisons. The BPI-Facial is chosen as a secondary outcome measure to address the unique distribution (e.g., trigeminal nerve regions) and complex characteristics (e.g., burning sensation, allodynia) of cephalo-facial PHN. BPI-Facial enables multidimensional evaluation of EA’s comprehensive effects, including pain intensity, quality, and interference with emotions, sleep, and daily functioning. This complements the limitations of VAS by providing a broader perspective on treatment efficacy. Additionally, analyzing pain-related quality-of-life impairments helps verify the potential mechanisms through which EA improves overall health status. The PSQI is included as a secondary outcome to systematically assess the impact of pain on sleep architecture. Cephalo-facial PHN often causes severe sleep disturbances due to pain, and PSQI quantifies the indirect sleep-improving effects of EA via pain relief. It also elucidates the interrelationships between sleep recovery, pain reduction, and quality-of-life enhancement, offering indirect evidence for EA’s multi-target regulatory mechanisms. The SDS and HAMD are selected as secondary measures to rapidly screen for depressive symptoms induced by chronic pain. These tools quantify the indirect psychological benefits of EA through somatic symptom relief (e.g., pain) and reveal the bidirectional relationship between pain and depression. This is particularly relevant for cephalo-facial PHN patients, who are prone to comorbid moderate-to-severe depression due to prolonged pain. SDS and HAMD provide supplementary evidence for EA’s “holistic” (somatic-psychological) efficacy and strengthen the comprehensive assessment of health outcomes. The SF-36 is adopted as a secondary outcome to evaluate multidimensional health states, including physiological, psychological, and social functioning. It quantifies the overall quality-of-life improvements resulting from pain relief and systematically reflects EA’s comprehensive effects on daily activity capacity, emotional well-being, and social participation across its eight domains. This provides standardized evidence for the sequential efficacy of EA (“pain relief-functional recovery-quality-of-life enhancement”) and enhances the quantitative assessment of holistic health benefits.

Overall, our research findings will enhance the global comprehension of the clinical significance and effectiveness of EA in managing PHN. Consequently, this may pave the way for EA to emerge as a viable, low-side-effect treatment option for PHN patients, aiming to mitigate their neuropathic pain, alleviate skin hypersensitivity, and ultimately elevate their quality of life and facilitate their work involvement.

## Limitations

This study has limitations due to the inherent flaws in the blinded implementation of acupuncture clinical trials. Despite our efforts to minimize potential bias by keeping statisticians and assessors blinded, operator knowledge of grouping is indeed unavoidable due to the nature of electroacupuncture treatment. In addition, patients may disrupt the blinding due to differences in treatment modalities, which may introduce bias into the study results. To minimize the interference of blinding limitations on the trial results, we will evaluate the blinding of operators and patients at the end of the trial, including operator-knowledge of the grouping, patient-perceived differences in treatments, and other factors that bias the blinding, such as differences in communication between operators and patients in different groups. In the meantime, we have referred to the relevant literature on similar studies (59, 60), and if bias occurs, we will assess the possible impact of each source of bias on the study results to estimate the extent of potential placebo effects and other biases. It is hoped that future studies will have better ways to address these limitations, and we will continue to monitor this issue.

Additionally, patients are required to visit the clinic on a weekly basis for three acupuncture sessions, each treatment takes more than an hour, the cost of time is greatly increased, and it is not as convenient as taking medication at home. For patients who face difficulties in frequently visiting the clinic, patient-self electroacupuncture treatment can be considered under the supervision of trained medical professionals. This form of treatment offers advantages such as enhanced convenience and personalized adjustments, aiding patients in their long-term management and rehabilitation. However, it also poses challenges including operational risks, variations in efficacy, substantial training requirements, and regulatory difficulties (61, 62). In our future research, we will delve deeper into the potential benefits and challenges associated with patient-self electroacupuncture.

## Conclusion

In summary, this research protocol is a multicenter, randomized controlled trial with blinding of both patients and assessors. The objectives are to evaluate the effectiveness and safety of EA compared with medication; and to clarify whether EA in the treatment of cephalo-facial PHN has a clear and stable advantage over oral pregabalin alone in terms of improvement in mood, quality of sleep, and quality of life. This finding will provide new scientific and effective ideas for the clinical treatment of PHN.
